# Research on the Strategy of Motion Constraint-Aided ZUPT for the SINS Positioning System of a Shearer

**DOI:** 10.3390/mi8110340

**Published:** 2017-11-22

**Authors:** Hai Yang, Wei Li, Tao Luo, Chengming Luo, Haibo Liang, He Zhang, Yaxiong Gu

**Affiliations:** 1 School of Mechatronic Engineering, Southwest Petroleum University, Chengdu 610500, China; yanghaicumt@163.com (H.Y.); luotao618@163.com (T.L.); hyj2643446@126.com (H.L.); zhanghe@swpu.edu.cn (H.Z.); guyxswpu@163.com (Y.G.); 2 School of Mechatronic Engineering, China University of Mining and Technology, Xuzhou 221116, China; 3 College of Internet of things Engineering, Hohai University, Changzhou 213022, China; luocm@hhu.edu.cn

**Keywords:** shearer, strap-down inertial navigation system, positioning error calibration, motion constraint-aided, zero velocity updated

## Abstract

The accurate measurement of position and orientation for shearers is a key technology in realizing an automated, fully-mechanized, coal mining face. Since Global Positioning System (GPS) signal cannot arrive at the coal mine underground, wireless sensor network positioning system cannot operate stably in the coal mine; thus a strap-down inertial navigation system (SINS) is used to measure the position and orientation of the shearer. Aiming at the problem of the SINS accumulative error, this paper proposes a positioning error correction method based on the motion constraint-aided SINS zero velocity updated (ZUPT) model. First of all, a stationary state detection model of the shearer is built with median filter based on the acceleration and angular rate measured by the SINS. Secondly, the motion of the shearer is analyzed using coal mining technology, then the motion constraint model of the shearer is established. In addition, the alternate action between the motion constraint model and the ZUPT model is analyzed at the process of movement and cessation of the shearer, respectively; hence, the motion constraint-aided SINS ZUPT model is built. Finally, by means of the experimental platform of the SINS for the shearer, the experimental results show that the maximum position error with the positioning model proposed in this paper is 1.6 m in 180 s, and increases by 92.0% and 88.1% compared with the single motion constraint model and single ZUPT model, respectively. It can then restrain the accumulative error of the SINS effectively.

## 1. Introduction

Coal is the principal basic energy and raw material in China. To meet the requirements for the development of the national economy, coal has been considered to be the main energy source for a long time to come [1]. The greater the demand for coal, the more frequently security incidents in the coal mine will occur. Hence, the safe and efficient exploitation of the coal mine has become a key research area for many scholars [2]. In the coal exploitation field, the notion that the mechanization and automation of mining equipment are basic factors for less-manned or unmanned mining has gained widespread acceptance. Furthermore, the precise location-awareness of the shearer is one of the key technologies for the automation of mining machines [3].

**Figure 1:**
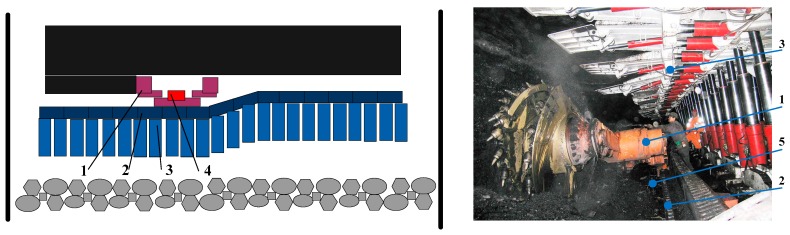
A diagram for the “three-machine” of a fully-mechanized coal face and its realistic scenario. 1—Shearer; 2—conveyer scraper; 3—hydraulic support; 4—SINS; and 5—central trough.

The “three-machine” of a fully-mechanized coal face includes the shearer, scraper conveyer, and hydraulic support. Among of them, the shearer crushes the coal, then the conveyer scraper moves the coal which is broken by the shearer. Meanwhile, the hydraulic supports sustain the top of the mine tunnel. Moreover, there are over a hundred sets of hydraulic supports, which placed along the work face. As a consequence, they coordinate each other closely and achieve the exploitation of coal. For the fully-mechanized coal face, the shearer works close to the coal wall. Furthermore, the body of the shearer rides the central trough of the scraper conveyer [4]. Using the traction device of the shearer, the shearer can move to-and-fro along the central trough of the scraper conveyer, and then it cuts a row of coal. In this case, the scraper conveyer is placed along the coal wall and connected with every hydraulic support through advancing rams. Consequently the hydraulic support can sustain the roof at the coal gab area [5]. A diagram of the “three-machine” of a fully-mechanized coal face and its real scenario are shown in Figure 1. It is obvious that a coordinated “three-machine” can be developed into an automatic operation. However the position, velocity, and orientation of the shearer often restricts the operation of the hydraulic support and scraper conveyer. As a consequence, in order to achieve coordinated operation of the “three-machine”, it is meaningful to measure the position and orientation of the shearer accurately.

In order to measure the accurate position and orientation of the shearer, some scholars proposed a strap-down inertial navigation system (SINS). The SINS, which integrates the accelerometer and gyroscope readings computes the position and attitude of the shearer without any external aids after the required initialization and alignment [6,7]. Therefore, considerable research effort has been focused on this topic recently. Reid et al. [8] described recent advances in the development of an integrated inertial guidance system for the automation of the longwall coal mining process and represents a breakthrough in achieving practical and reliable automated face alignment. Fan et al. [9] proposed a strap-down inertial navigation system for dynamic shearer positioning and established a shearer inertial navigation model. However, a major problem with this kind of solution is that the estimation error grows with time, due to the typical drift of the inertial measurement, because of the poor working conditions for the shearer in the coal mine. Since the measured accuracies of the accelerometer and gyroscope in the SINS are limited by the high cost of the sensors, the measured error of the SINS will cause the accumulative error in the calculation process of the orientation and position [10]. Hence, the positioning accuracy of the SINS for the shearer will be significantly influenced.

Nowadays with the rapid development of technology, some high-accuracy inertial measurement units (IMUs) have been developed by many scholars, such as the electrostatic suspended gyroscope (ESG), ultrasonic suspension gyroscope, and other gyroscopes [11]. In consideration of the SINS reliability for the poor working condition and production costs of coal exploitation, the IMU with micro-electro-mechanical system (MEMS) technology is still used by most engineers in actual coal mine applications. MEMS is the technology of microscopic devices, particularly those with moving parts. MEMS are made up of components between 1 and 100 μm in size, and MEMS devices generally range in size from 20 μm to 1 mm. Accelerometers made with MEMS technology and have the advantages of small size, light weight, low energy consumption, and so on. The frequently-used micro-accelerometers are of the silicon piezoresistive type, capacitive type, twisting type, and other types. In addition, MEMS gyroscopes measure the angular rate of a mobile target based on the Coriolis force which is a tangential force of a rotating object.

In addition, MEMS IMUs have great reliability and low-cost [12,13,14,15,16]. Consequently, the measured errors of the gyroscopes and accelerometers will be brought to the positioning result in a process of position calculation. Due to the drift of low-cost MEMS sensors and recursive computation, Rui et al. [17] proposed a zero velocity update (ZUPT) method for pedestrian tracking based on foot-mounted inertial sensors suffering from accumulative velocity and position errors. However, it is a known fact that ZUPTs cannot reduce all errors, especially as the heading error is not observable. Muhammad et al. [18] proposed a pedestrian gait positioning method using motion constraints and any other valuable heading reduction information. Nevertheless, the above scholars just focus on pedestrian localization and have not researched the fusion method between ZUPT and motion constraints.

Aiming at the above problems, this paper analyzes the motion characteristics of the shearer and proposes a measured error correction strategy for the SINS using ZUPT and motion constraint methods. Firstly, with full consideration of the motion constraint characteristic of the shearer in a fully-mechanized working face, the ZUPT model can be built with a Kalman filter (KF) based on the transient stationary process of the shearer, according to the stationary state detection algorithm. Secondly, the shearer moves along with the scraper conveyer and cuts the coal in the normal working condition. As a consequence, the motion of the shearer is restricted by the scraper conveyer. In addition, the corrected position solution model of the SINS is built based on the motion constraint of the shearer. Finally, the motion constraint-aided SINS ZUPT positioning model of the shearer can be obtained.

## 2. Motion Characteristic Analysis for Shearer

**Figure 2:**
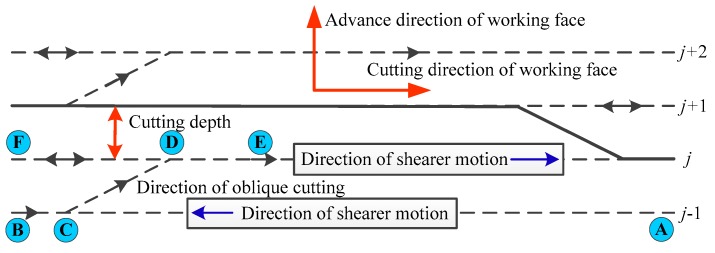
Schematic diagram of the shearer terminal oblique cutting.

The hydraulic supports are stood side by side in the fully mechanized coal mining face. Meanwhile, the scraper conveyer is placed along with cutting direction of working face and sits before the hydraulic supports. Additionally, every hydraulic support is connected to the scraper conveyer through a hydraulic cylinder which is used to push the scraper conveyer [19]. The shearer rides the scraper conveyer and moves iteratively along the scraper conveyer. The shearer cuts the coal. Meanwhile, the hydraulic supports are pulled by corresponding hydraulic cylinders. Afterwards the roof beams of hydraulic supports rise and push the roof of coal working face [20]. Hence, the hydraulic supports are fastened to the coal, then the hydraulic supports push the scraper conveyer. A diagram of the shearer motion path is shown in Figure 2. After the shearer finishes cutting an entire row of coal and moves from one side of the fully mechanized coal mining face to the other side, which likes from point A to point B in Figure 2, the hydraulic supports behind the shearer have already pushed scraper conveyer with a distance in the advance direction of working face. This distance is described as cutting depth of the fully mechanized coal mining face. As a consequence, the scraper conveyer forms an oblique line which is expressed as line CD in Figure 2, because of pushing from the hydraulic supports. After that shearer moves reversely and starts to cut a new row of coal along with that oblique line CD, then there is a stationary moment of the shearer. When the shearer passes the oblique line of scraper conveyer and arrives the straight area at point E, the remaining part of scraper conveyer will be pushed by hydraulic support and aligned with other part of scraper conveyer. Finally the shearer must move to point F and cut the coal at the end of working face. Meanwhile, the other stationary state of the shearer is emerged at this moment. Then the shearer can continue to cut the next row of coal along with the straight scraper conveyer. This process is called terminal oblique cutting of the shearer.

According to above analysis of the shearer motion characteristic, the shearer will change motion direction for three times. Consequently the shearer will be led to brief stop three times in the process of terminal oblique cutting. In addition, if shearer works at straight area of scraper conveyer, the shearer would be led to stop traction inevitably because of the various enrichment forms of coal and the disconnected operation of miner. Hence, the acceleration and angular rate of the shearer measured by SINS can be applied to detect the stationary state of the shearer real-time. As a consequence, the positioning error of the SINS can be corrected by ZUPT method real-time.

The shearer always moves along with the scraper conveyer at the working process. So the vertical velocity and lateral velocity of the shearer can be regarded as two random noises which close to zero, if the slight lateral movement of the shearer is not considered. Hence, based on this rule, the positioning performance of the SINS for shearer can be restricted and corrected in the movement process of the shearer. Consequently, above rule, called as the motion constraint of the shearer, can be used to assist and compensate the ZUPT method effectively.

## 3. Stationary State Detection for the Shearer

According to Newton’s law, the acceleration and angular rate of one object can express its motion state directly. For example, if both the acceleration and angular rate of one object are zero, it will stay in its state of rest or of uniform motion in a straight line [21]. However, the shearer cannot move with uniform motion in a straight line based on a near-zero acceleration and angular rate, because of the shearer mechanical vibration and low-accuracy speed control from the traction motor. As a consequence, if the shearer remains in a stationary state, the acceleration vector measured by the SINS will be close to the gravitational acceleration, denoted as *g* = 9.81 m/s^2^. Meanwhile, the angular rate measured by the SINS will be close to zero. According to this property, the vector norm of the three-axis acceleration and angular rate from the SINS can be described as follows:

(1) $$\{\begin{array}{c} ∥a_{k}^{b}∥=\sqrt{{\left(a_{x,k}^{b}\right)}^{2}+{\left(a_{y,k}^{b}\right)}^{2}+{\left(a_{z,k}^{b}\right)}^{2}}\\ ∥\omega_{ib,k}^{b}∥=\sqrt{{\left(\omega_{x,k}\right)}^{2}+{\left(\omega_{y,k}\right)}^{2}+{\left(\omega_{z,k}\right)}^{2}} \end{array}$$

where $∥a_{k}^{b}∥$ and $∥\omega_{ib,k}^{b}∥$ represent the vector norms of the acceleration and angular rate, respectively. $a_{k}^{b}={\left[a_{x,k}^{b},a_{y,k}^{b},a_{z,k}^{b}\right]}^{T}$ is the acceleration vector in the body coordinate frame (b-frame) which is measured by the SINS. $\omega_{ib,k}^{b}={\left[\omega_{x,k},\omega_{y,k},\omega_{z,k}\right]}^{T}$ is the angular rate vector which is measured by the SINS. *k* represents the time index with a corresponding period *T*, and $T=t_{k+1}-t_{k}$.

The two judgment thresholds of acceleration are defined as $S_{a,\max}$ and $S_{a,\min}$ which must meet $S_{a,\max}>g>S_{a,\min}$. Meanwhile, a judgment of the angular rate is defined as $S_{\omega }$. By judging whether $∥a_{k}^{b}∥$ and $∥\omega_{ib,k}^{b}∥$ meet their thresholds, at every moment the acceleration and angular rate of the SINS are divided into two states which contain a stationary state and a non-stationary state. According to a large number of experimental tests, $∥a_{k}^{b}∥$ must be in the range of two predefined thresholds $S_{a,\max}$ and $S_{a,\min}$:

(2) $$C_{1}=\{\begin{array}{l} 1,\;S_{a,\max}>∥a_{k}^{b}∥>S_{a,\min}\\ 0,\;\mathrm{Others} \end{array}$$

$∥\omega_{ib,k}^{b}∥$ must be smaller than a predefined threshold $S_{\omega }$:

(3) $$C_{2}=\{\begin{array}{l} 1,\;∥\omega_{ib,k}^{b}∥<S_{\omega }\\ 0,\;\mathrm{Others} \end{array}$$

where $C_{1}$ and $C_{2}$ are the judgment results of the stationary state for acceleration and the angular rate, respectively. Only when both $C_{1}$ and $C_{2}$ are one, can the shearer be considered to stay within a stationary state at this moment. Hence, it is expressed as:

(4) $$C_{i}=C_{1}\cap C_{2}$$

However, because of the influence from the measured noise of acceleration and angular rate, some erroneous judgments will emerge for the logic calculation of the stationary state. Consequently, the erroneous judgments in $C_{i}$ are defined as salt-and-pepper noise. 

**Figure 3:**
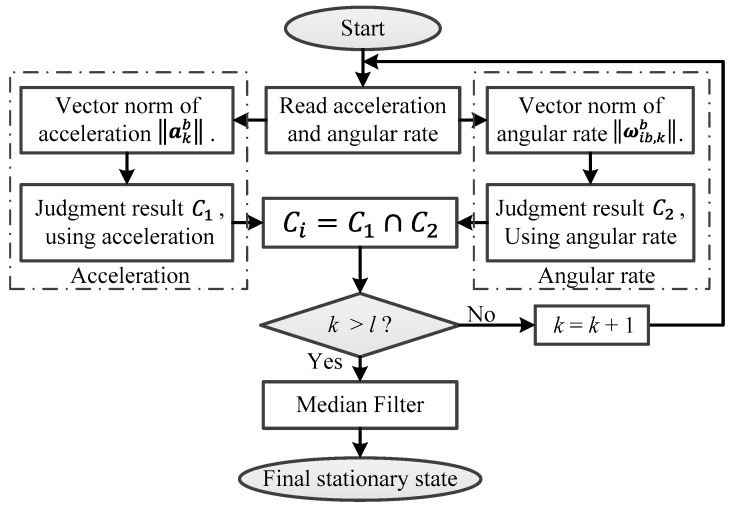
Flowchart for the judgment of the strap-down inertial navigation system (SINS) stationary state.

A median filter is widely used to denoise salt-and-pepper noise in image processing. The median filter is a nonlinear signal processing technology based on the order statistical theory, which can effectively suppress the noise. The basic principle of the median filter is that a point in the numerical sequence is replaced by the median value of each point in a neighborhood. Then, the numerical sequence is closer to the real value and some isolated noise points can be removed [22]. For this paper, the median filter is used to smooth $C_{i}$, and then the salt-and-pepper noise in $C_{i}$ can be removed and the stable judgment result of the stationary state can be obtained. This is defined as $C_{m,i}$:

(5) $$C_{m,i}=f_{\mathrm{medianfilter}}(C_{i},l)$$

where *l* is the window length of the median filter. The flowchart for the judgment of the SINS stationary state is shown in Figure 3.

## 4. Motion Constraint-Aid ZUPT Model of the Shearer

### 4.1. State Equation of the Shearer Motion Model

The ZUPT strategy for the SINS is mainly that the velocity error, as a measurement vector, is used to correct the positioning value based on the zero-velocity characteristic for the stopped shearer [23]. According to the stationary state detection result in Section 3, the ZUPT of the SINS can be achieved more effectively. 

According to Newton’s law and the SINS calculation model, the position calculation model of the shearer can be obtained and it is expressed as:

(6) $$\{\begin{array}{c} P_{k+1}^{n}=P_{k}^{n}+V_{k}^{n}T+\left(C_{b}^{n}a_{k}^{b}-g^{n}\right)T^{2}/2\\ V_{k+1}^{n}=V_{k}^{n}+\left(C_{b}^{n}a_{k}^{b}-g^{n}\right)T \end{array}$$

where, $P_{k}^{n}$ is the position vector of the shearer in the navigation frame (n-frame) and $V_{k}^{n}$ is the velocity vector of the shearer in the n-frame. $a_{k}^{b}$ is the acceleration vector of the shearer measured by the SINS in the body frame (b-frame). $C_{b}^{n}$ is a transformation matrix from the b-frame to the n-frame. $g^{n}={\left[\begin{array}{ccc} 0 & 0 & g \end{array}\right]}^{T}$ is the gravity vector in the n-frame and *T* is the sampling period of the SINS. Equation (6) is calculated with total differential equation, then the calculation result is expressed as:

(7) $$\{\begin{array}{c} \delta P_{k+1}^{n}=\delta P_{k}^{n}+\delta V_{k}^{n}T-\left(C_{b}^{n}a_{k}^{b}\times \right)\frac{T^{2}}{2}\delta A_{k}+C_{b}^{n}\frac{T^{2}}{2}\delta a_{k}^{b}\\ \delta V_{k+1}^{n}=\delta V_{k}^{n}-\left(C_{b}^{n}a_{k}^{b}\times \right)T\delta A_{k}+C_{b}^{n}T\delta a_{k}^{b} \end{array}$$

where $\delta P^{n}$ and $\delta V^{n}$ are the position error and velocity error vectors of the shearer in the n-frame, respectively. δA is the attitude error vector and $\delta a^{b}$ is the measured noise of accelerometer in SINS. ***a***^*b*^ × represents the anti-symmetric matrix of acceleration vector $a^{b}$.

As a consequence, the state vector of the shearer positioning system is defined as:

(8) $$x_{k}={\left[\begin{array}{ccc} \delta P_{k}^{n} & \delta V_{k}^{n} & \delta A_{k} \end{array}\right]}^{T}$$

Then Equation (7) can be expressed as:

(9) $$x_{k+1}=F_{k,k+1}x_{k}+G_{k}W_{k}$$

where $W_{k}$ is the system noise vector of the state equation and $W_{k}={\left[\begin{array}{cc} \omega_{\varepsilon } & \delta a^{b} \end{array}\right]}_{k}^{T}$. $\omega_{\varepsilon }$ is the measured noise of the gyroscope in the SINS. In addition $G_{k}$ is the one-step transition disturbance matrix of system, and $F_{k,k+1}$ is the one-step transition matrix. Namely:

(10) $$F_{k,k+1}={\left[\begin{array}{ccc} I_{3\times 3} & T\;\cdot \;I_{3\times 3} & -\left(C_{b}^{n}a^{b}\times \right)T^{2}/2\\ 0_{3\times 3} & I_{3\times 3} & -\left(C_{b}^{n}a^{b}\times \right)T\\ 0_{3\times 3} & 0_{3\times 3} & I_{3\times 3} \end{array}\right]}_{k}$$

(11) $$G_{k}={\left[\begin{array}{cc} 0_{3\times 3} & C_{b}^{n}T^{2}/2\\ 0_{3\times 3} & C_{b}^{n}T\\ C_{b}^{n} & 0_{3\times 3} \end{array}\right]}_{k}$$

### 4.2. Zero Velocity Updated Model of the SINS

Since the velocity calculated by the SINS is equal to the velocity error of the positioning system for the stopped shearer, the measurement equation of the shearer SINS ZUPT model can be built. Namely:

(12) $$V_{k}^{n}=H_{ZUPT,k}x_{k}+\upsilon_{k}$$

where $H_{ZUPT,k}=\left[\begin{array}{ccc} 0_{3\times 3} & I_{3\times 3} & 0_{3\times 3} \end{array}\right]$, and $\upsilon_{k}$ is the measurement noise of the SINS.

Consequently, according to the state space equation composed by Equation (9) and Equation (12), the SINS positioning error of the shearer can be corrected by the ZUPT model using a Kalman filter [24] and the stationary state detection algorithm. Then, the SINS ZUPT model of the shearer is achieved.

### 4.3. Motion Constraint-Aided ZUPT Algorithm of the Shearer

During the process of the SINS localization, the ZUPT strategy of the SINS can be realized based on the stationary state detected result of the shearer. Then, the positioning accuracy of the SINS for the shearer would be increased. However, there are many dynamic error sources in the process of the actual shearer localization; the normal ZUPT strategy is almost restricted by a time interval of when the shearer stops working. Hence, it is difficult to achieve the ideal positioning performance for the shearer under the actual working condition.

According to the above analysis, the shearer moves along with the coal mine working face, which is restricted by the scraper conveyer. Then, the velocity constraint characteristic of the shearer can assist the ZUPT algorithm of the SINS. Consequently, the SINS motion constraint-aided ZUPT strategy of the shearer can be achieved.

**Figure 4:**
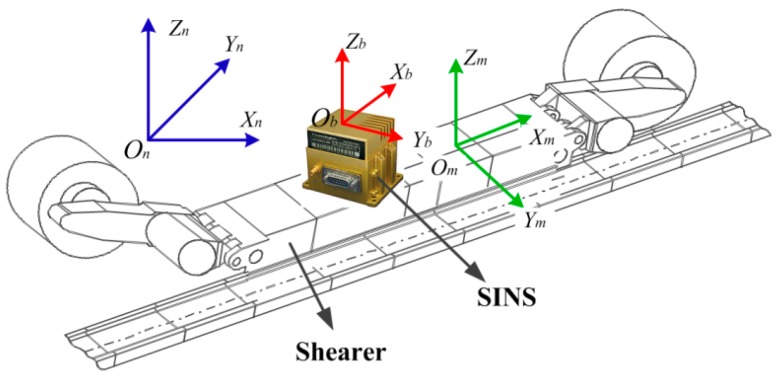
The relationship for different coordinates of the shearer.

First of all, a rectangular coordinate system, which is fixed on the shearer body, is defined as the machine frame (m-frame) of the shearer. The three axes of the m-frame point forward, laterally, and vertically with respect to the shearer, while the b-frame is fixed on the SINS. The relationship among the m-frame of the shearer, the b-frame of the SINS, and the n-frame is indicated in Figure 4. In addition, the SINS is fixed to the shearer. Due to the finite installation accuracy of the SINS, there is an installation angle between the m-frame of the shearer and the b-frame of the SINS. The installation angle is expressed as $\beta^{m}=\left[\begin{array}{ccc} \beta_{x}^{m} & \beta_{y}^{m} & \beta_{z}^{m} \end{array}\right]$. Hence, the velocity of the shearer in the m-frame is represented as:

(13) $$V^{m}=C_{b}^{m}C_{n}^{b}V^{n}$$

where $C_{b}^{m}$ is the attitude transfer matrix from the b-frame to the m-frame.

Equation (13) is calculated with a total differential equation, and the calculation result is expressed as:

(14) $$\delta V^{m}=C_{b}^{m}\left(C_{n}^{b}\delta A\times V^{n}+C_{n}^{b}\delta V^{n}\right)+\delta \beta^{m}\times C_{b}^{m}C_{n}^{b}V^{n}$$

where $\delta \beta^{m}$ is a vector of the installation angle deviation of the SINS for the shearer.

The state vector of the shearer $x_{k}$ is combined with $\delta \beta^{m}$, then the new state vector of the shearer can be obtained as $x_{s,k}={\left[\begin{array}{cc} x_{k}^{T} & \delta \beta^{m} \end{array}\right]}^{T}$. Since the SINS is fixed to the shearer, the installation angle deviation of the SINS does not change as time passes. Hence, it can be obtained as:

(15) $$\delta \beta_{k+1}^{m}=\delta \beta_{k}^{m}+w_{\beta ,k}$$

where $w_{\beta ,k}$ is the transmitted noise of the SINS installation angle deviation, which is the Gauss white noise with zero mean. According to the state equation of Equation (9), the new state equation which includes the $\delta \beta^{m}$ can be expressed as:

(16) $$\underset{x_{s,k+1}}{\underbrace{\left[\begin{array}{c} x_{k+1}\\ \delta \beta_{k+1}^{m} \end{array}\right]}}=\underset{F_{s,k,k+1}}{\underbrace{\left[\begin{array}{cc} F_{k,k+1} & 0_{9\times 3}\\ 0_{3\times 9} & I_{3\times 3} \end{array}\right]}}\underset{x_{s,k}}{\underbrace{\left[\begin{array}{c} x_{k+1}\\ \delta \beta_{k+1}^{m} \end{array}\right]}}+\underset{G_{s,k}}{\underbrace{\left[\begin{array}{cc} G_{k} & 0_{9\times 3}\\ 0_{3\times 9} & I_{3\times 3} \end{array}\right]}}\underset{W_{s,k}}{\underbrace{\left[\begin{array}{c} W_{k}\\ w_{\beta ,k} \end{array}\right]}}$$

According to Equation (14), the measurement equation of the SINS is obtained based on the motion constraint of the shearer. It is expressed as:

(17) $$\underset{V_{MC,k}^{m}}{\underbrace{\left[\begin{array}{c} V_{x}^{m}\\ V_{z}^{m} \end{array}\right]}}=\underset{H_{MC,k}}{\underbrace{\left[\begin{array}{ccc} 1 & 0 & 0\\ 0 & 0 & 1 \end{array}\right]\left[\begin{array}{cccc} 0_{3\times 3} & C_{b}^{m}C_{n}^{b} & -C_{b}^{m}C_{n}^{b}\left(V^{n}\times \right) & \left(-\left(C_{b}^{m}V^{b}\right)\times \right) \end{array}\right]}}x_{s,k}+\upsilon_{v^{m},k}$$

where $\upsilon_{v^{m},k}$ is the measured noise of the motion constraint equation and $V_{MC,k}^{m}$ is the shearer velocity vector with the motion constraint.

The measurement equation of the SINS ZUPT model is combined with that of the shearer motion constraint model, and then the measurement equation of whole SINS positioning system can be obtained as:

(18) $$\{\begin{array}{c} V_{k}^{n}=H_{ZUPT,k}x_{k}+\upsilon_{k},\;C_{m,k}=1\\ V_{MC,k}^{m}=H_{MC,k}x_{s,k}+\upsilon_{v^{m},k},\;C_{m,k}=0 \end{array}$$

**Figure 5:**
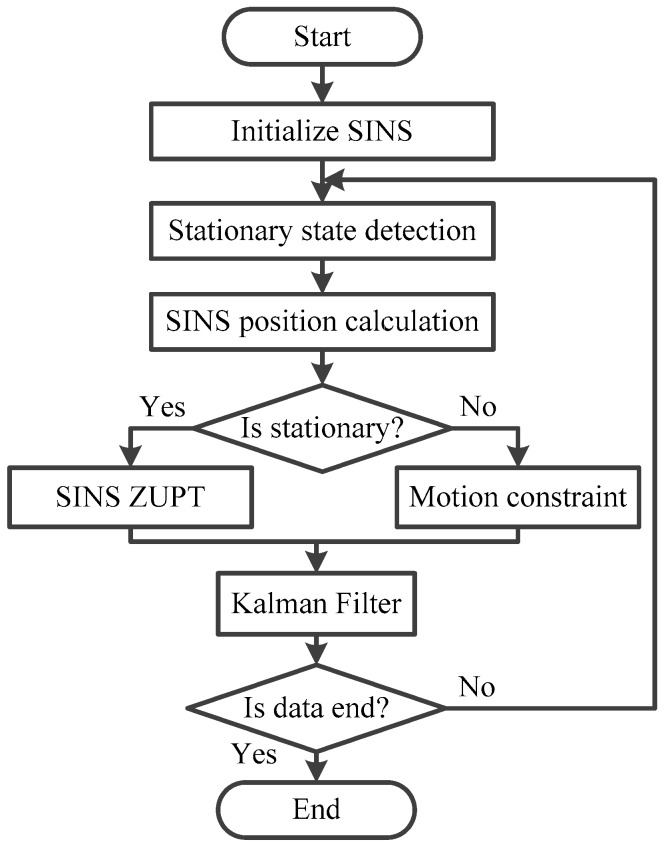
Flowchart of the shearer positioning model with the motion constraint-aided sins zero velocity updated (ZUPT) method.

According to the stationary state detection result for the shearer, the automatic mutual switch operation between the ZUPT model and the motion constraint model can be realized. Then the accumulative error of the SINS will be corrected effectively using the Kalman filter (KF) algorithm [25] and the motion constraint-aided SINS ZUPT model for the shearer is finished. In addition, the flowchart of the shearer positioning model with the motion constraint-aided SINS ZUPT method is shown in Figure 5.

## 5. Experimental Study

In order to evaluate the positioning performance of the motion constraint-aided SINS ZUPT model for the shearer proposed in this paper, a shearer model in the basement is applied to simulate the actual shearer in a fully-mechanized coal mining face. Additionally, an experimental platform of the SINS positioning system for the shearer model is built, and then the experimental verification for positioning model can be implemented successfully.

### 5.1. Construction of the Experimental Platform

In a fully-mechanized mining face, the shearer rides the scraper conveyer and moves along with the working face. In view of the special environment of the coal mine working face, it is difficult to conduct a field experiment of the shearer using the SINS positioning platform. Consequently, a shearer model in the laboratory has to be used to test via simulation experiments. Meanwhile, considering that the shearer works in dreadful conditions, in order to simulate its working conditions, the shearer model is placed at an underground car park conduct the experiments. As a consequence, the experimental environment is closer to the actual working condition of the shearer.

The SINS is installed on the shearer and supplied by a mobile power bank. Meanwhile, the inertial data is transferred with two Bluetooth modules. One Bluetooth module is connected to the SINS with an RS232 ribbon cable, and the other is connected to the computer by a wired USB serial connection (Bluetooth 1.1 and USB 2.0). Then the computer can collect the inertial data of the SINS in real-time based on the wireless Bluetooth communication. 

**Figure 6:**
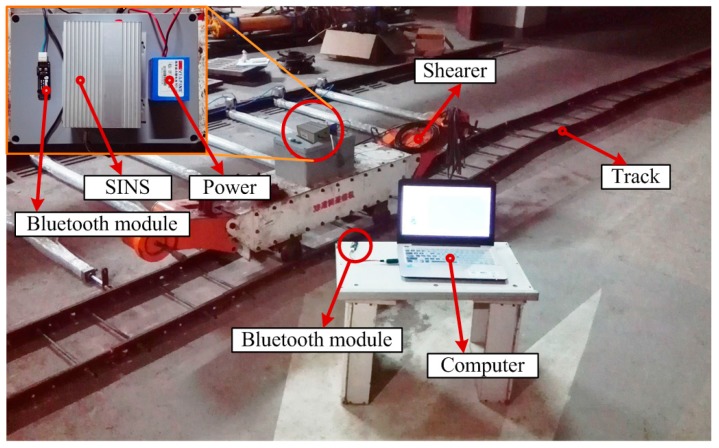
Experimental scene of the shearer for the SINS positioning system.

The used SINS includes a six degree-of-freedom MEMS IMU which consists of a triaxial accelerometer and a triaxial gyroscope. The main parameters of the MEMS accelerometer and gyroscope are described in Table 1. The baud rate of the SINS is 115,200 bit/s, and the sampling period is 0.01 s. The attitude reference precision of the SINS is 10″, and the accuracy of the gravitational acceleration is not less than 2 × 10^−5^ *g*. The angular rate resolution of the gyroscope is 0.025°/s. The RS232 serial communication is used only for data transmission between the SINS and the computer. The maximum transmission baud rate of the Bluetooth model is 1,382,400 bits/s. The maximum received distance between the two Bluetooth modules is up to 100 m in ideal conditions (free space). Figure 6 shows the experimental scene of the shearer SINS positioning system.

The shearer model moves along the track which is laid on the floor in advance, while noting that the motion trajectory of the shearer model forms a rectangle. Additionally, in the movement process, the shearer model will be stopped randomly. For the first experiment, the shearer model moves in a circle and the experiment lasts for 180 s.

### 5.2. Experimental Result

In the process of experiment, the SINS measures the acceleration and angular rate of the shearer in real-time. After setting the initial value of the SINS, the acceleration and angular rate of the SINS can be used to calculate the attitude and position based on the motion constraint-aided ZUPT positioning model. Then the accurate position of the shearer will be obtained.

**Figure 7:**
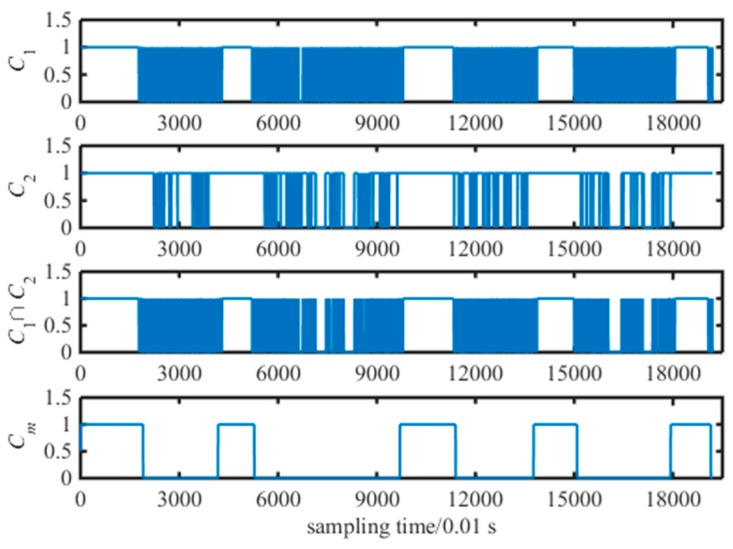
Detection result of the stationary state of the shearer.

Firstly, according to the stationary state detection model of the shearer in Section 3, the judgment thresholds of the stationary state detection model is set based on the acceleration and angular rate of the SINS. Then the moment of the shearer stationary state can be obtained by median filter algorithm. Hence, the detection result of the shearer stationary state is plotted in Figure 7. From this figure, the first sub-figure and second sub-figure are expressed as *C*_1_ and *C*_2_ from Equations (2) and (3), respectively. Due to the measurement noise in the acceleration and angular rate, the misjudgment for a stationary state, described as salt-and-pepper noise, will emerge, when the integrated detection result *C_i_* for a stationary state is calculated. As a consequence, the misjudgment of the stationary state is calculated by the median filter, and the result is shown as fourth sub-figure in Figure 7. From this sub-figure, the stationary state detection result after median filter can stably reflect the moment of the shearer parking state effectively. Meanwhile, the misjudgment caused by measured noise is already filtered out. The results of the shearer stationary state can lay the foundation for the SINS solution.

**Figure 8:**
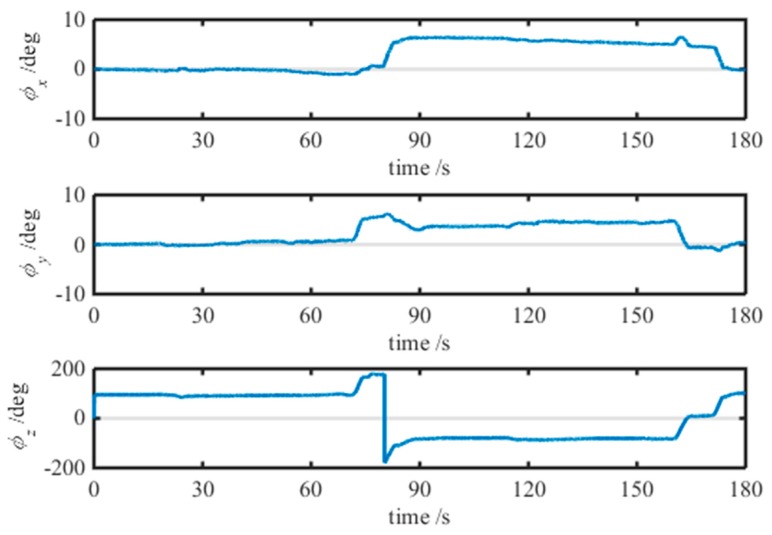
Result of the attitude solution for the SINS.

After the stationary state detection of the shearer, the three-axis attitude angles of the SINS are calculated by the quaternion solution model using the three-axis angular rates measured by the SINS. The attitude solution result which includes roll, pitch and yaw angles is plotted in Figure 8. From this figure, the pitch and roll angles show small, regular changes within 10°, and the yaw angle is changed from −180° to +180°, and clearly shows the shearer motion trajectory as a circle.

**Figure 9:**
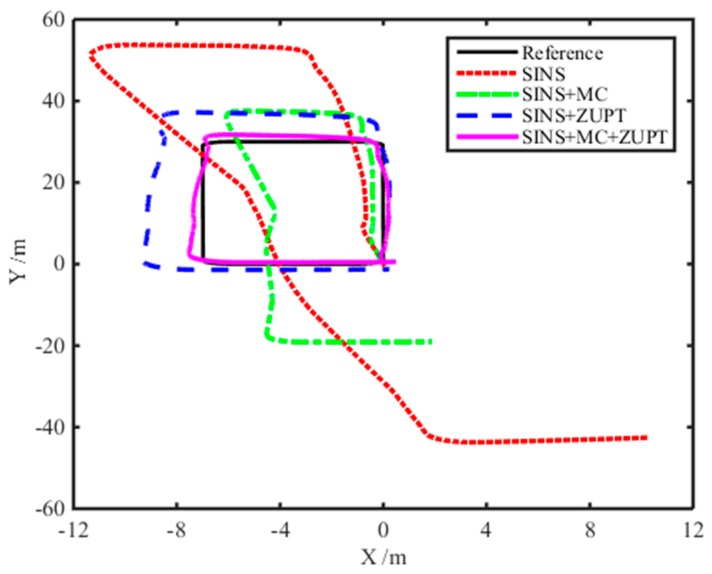
Positioning result of the SINS with different correction methods.

After the attitude solution of the SINS, the SINS positioning result is calculated by the SINS velocity and position solution model. Meanwhile, the result is optimized and corrected, with the motion constraint-aided SINS ZUPT model. The positioning result is plotted in Figure 9. First of all, the acceleration and angular rate measured by SINS are calculated with the quaternion solution method. In addition, the positioning error of the SINS is corrected based on the motion constraint method. Two positioning trajectories of pure SINS and the motion constraint of the shearer are expressed as red dots and green dash dot lines, respectively. From this figure, the positioning result of the pure SINS solution produces the gross accumulative error at the beginning of the shearer positioning. Meanwhile, its positioning trajectory has seriously diverged from the reference trajectory. Hence, the maximum of the position error with the pure SINS is about 43 m. After the positioning system is corrected with a Kalman filter-based motion constraint model, the positioning result is shown as green dash dot line in Figure 9. We can see that the orientation of the SINS positioning system with the motion constraint can follow the orientation of the reference trajectory. In addition, the accumulative error of the motion constraint model is smaller than that of the pure SINS. However, the motion constraint algorithm of the shearer belongs to the non-holonomic constraint. As a consequence, the positioning trajectory of the shearer motion constraint model also produces certain accumulative errors after the start of the positioning system. The maximum of the position error with the motion constraint model is about 20 m and surpasses that of the pure SINS 51.2% improvement.

Since the parking state of the shearer always emerged in the process of motion, the velocity calculated by the SINS cannot drop to zero at the shearer parking state, which is influenced by the measured error and the calculated attitude error of the SINS. Meanwhile, it is obvious that the positioning trajectory of the SINS still keeps moving, while the shearer works in a stationary state. As a consequence, the SINS will produce a large accumulative error, and the positioning accuracy of the SINS will decrease. According to the shearer stationary state detection model and the SINS ZUPT model, the SINS positioning result is corrected only using the ZUPT model. Combined with the above shearer motion constraint model, the positioning performance of the SINS can be verified alone more effectively with the motion constraint or the ZUPT model, respectively. Hence, the positioning trajectory of the shearer with the SINS ZUPT model is expressed as a blue dashed line. From this figure, the positioning trajectory with the SINS ZUPT strategy can follow the reference more effectively at the beginning of the experiment. However, a larger positioning error emerges at the mid-term of the experiment because of the influence from the SINS accumulative error. Thanks to the velocity correction of the ZUPT model, the positioning trajectory of the SINS gradually approaches the reference trajectory at the later period of the experiment, then the positioning error of the SINS decreases. As a consequence, the maximum of the shearer position error with the SINS ZUPT strategy is about 13.5 m, and surpasses that of the pure SINS 68.6% improvement. Meanwhile, the positioning accuracy of the SINS ZUPT model is better than that of the shearer motion constraint model.

According to motion constraint-aided SINS ZUPT model of the shearer, the positioning result of the SINS is corrected using the combined motion constraint and ZUPT algorithms. The shearer positioning result with the motion constraint-aided SINS ZUPT model is plotted as a solid purple line in Figure 9. From this figure, the positioning trajectory of the shearer with the motion constraint-aided SINS ZUPT model is closer to the reference trajectory compared with that using the single motion constraint or single ZUPT models. Through the shearer stationary state detection algorithm, the SINS ZUPT strategy with the Kalman filter can restrain the velocity drift error of the shearer at the parking state. The accumulative error of the SINS positioning system can be decreased more productively.

**Figure 10:**
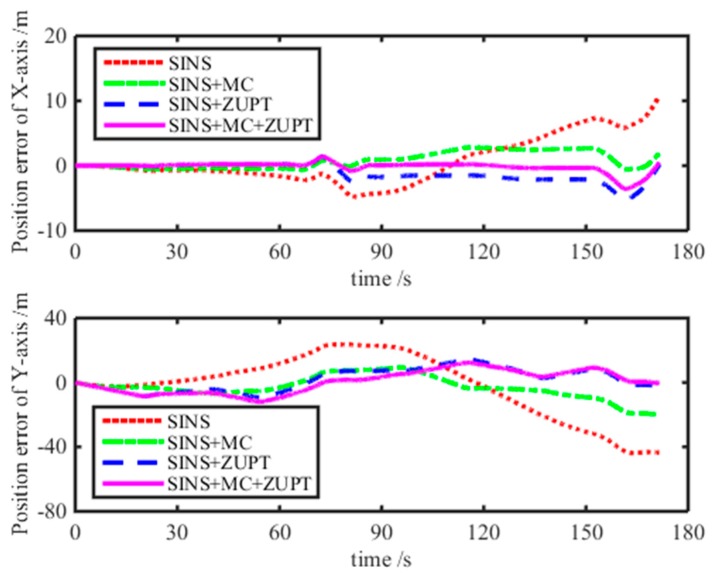
Position error of the SINS with different correction methods.

**Figure 11:**
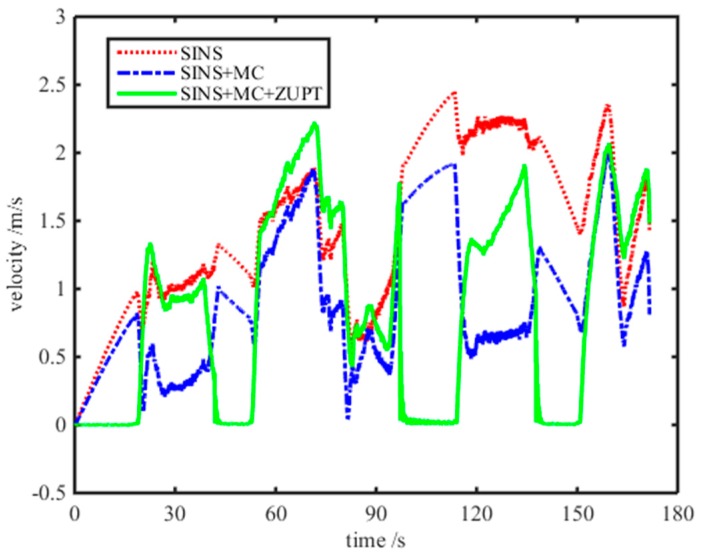
Velocity result of the SINS with different algorithms.

From Figure 9, the shearer positioning trajectory with the motion constraint-aided SINS ZUPT model can follow the reference trajectory more effectively. There is just a small position error at the second corner and the third corner of the reference trajectory. The maximum position error with the motion constraint-aided SINS ZUPT algorithm is just 1.6 m and decreases by 96.3% compared with the positioning error of the pure SINS. Meanwhile, its positioning accuracy increased by 92.0% and 88.1% compared with that with the single motion constraint model and single ZUPT model, respectively. Its stability of the SINS positioning system can be grown to a greater degree. The position error of the SINS positioning system with different algorithms is plotted in Figure 10. We can see that the positioning accuracy with the motion constraint-aided SINS ZUPT model surpasses that of the single motion constraint and single ZUPT models and has better stability. Moreover, the norm of the velocity vector for SINS calculated with different algorithms is shown in Figure 11. From this figure, we can see that the ZUPT strategy has reduced the velocity of the shearer to near-zero at the stationary state of the shearer, hence, the influence on positioning results from the velocity accumulative error has been reduced. Finally, positioning performance of the SINS positioning system is analyzed by four algorithms, such as the pure SINS solution model, single motion constraint model, single ZUPT model, and motion constraint-aided ZUPT model. Their analysis results are given in Table 2. From this table, the SINS positioning system of the shearer with the motion constraint-aided ZUPT model can restrain the accumulative error of the SINS effectively and has a high positioning accuracy, compared with [26].

**Figure 12:**
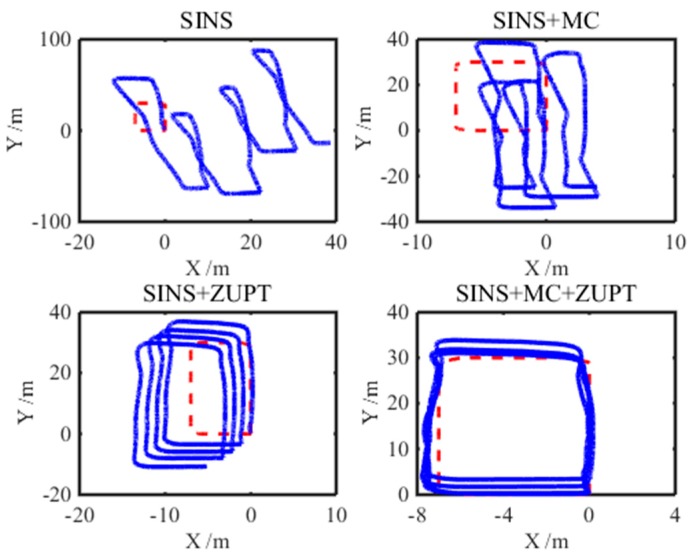
Experimental result of the SINS positioning system for the four-circle trajectory.

In order to verify the stability of the SINS positioning model proposed in this paper more effectively, the shearer model moved in four circles along the track and the experiment lasted for 718 s. In addition, the SINS positioning system is corrected by different correction models, and the experimental results are shown in Figure 12. First of all, from the first sub-figure, there is a very large accumulative error of the shearer positioning system for the pure SINS. Meanwhile, the positioning trajectory of the pure SINS has seriously strayed off the reference trajectory. Secondly, as can be seen from the second sub-figure, the SINS positioning trajectory with the single motion constraint model has a better convergence property than that of the pure SINS. As a consequence, the positioning error with the single motion constraint model is smaller than that with pure SINS. Thirdly, the experiment result of the SINS positioning system with the single ZUPT model is shown as the third sub-figure. As can be seen from this sub-figure, the shearer positioning error of the SINS with the single ZUPT model is much smaller than that with pure SINS. Moreover, the positioning trajectory with the single ZUPT model has better directivity than that in the second sub-figure. However, because of the SINS accumulative error, some drift of the positioning trajectory with the single ZUPT model already emerges. Finally, the SINS positioning system is corrected by the motion constraint-aided ZUPT model which is proposed in this paper. Its result is shown as the fourth sub-figure. From this sub-figure, the shearer positioning trajectory can follow the reference trajectory, when the shearer moves in four circles along the track. Meanwhile, the maximum of the SINS positioning system with the motion constraint-aided ZUPT model is about 2.4 m and is smaller than that with the single motion constraint and the single ZUPT models. As a consequence, for the longer experiment time, the SINS positioning system with the motion constraint-aided ZUPT model can follow the reference trajectory stably. Moreover, its positioning accuracy surpasses that with the single motion constraint and single ZUPT models.

## 6. Conclusions

Aimed at the SINS positioning system for low measured accuracy, the measured characteristics of the accelerometer and gyroscope in the SINS are analyzed. Meanwhile, the stationary state detection algorithm of the SINS is proposed based on the median filter. On this basis, the ZUPT model of the shearer is researched at the stationary state. Moreover, the motion constraint property of the shearer is analyzed. Then the motion constraint-aided SINS ZUPT model of the shearer is proposed in this paper. As a consequence, this method is used to correct the accumulative error of the SINS.

In order to evaluate the error correction method which is proposed in this paper, a shearer model in the basement is applied to conduct some experiments regarding SINS localization. Firstly, the shearer model moves in a circle along with the track; then the experimental result shows that the shearer positioning system with the pure SINS produces a large error at the beginning of the experiment. Moreover, the positioning error increases gradually as time goes by. Meanwhile, the SINS positioning error with the single motion constraint model is smaller than that of the pure SINS. In addition, the SINS positioning error with the single ZUPT model is also smaller than that of the pure SINS. However, the positioning system with both the single motion constraint and ZUPT models still produces large position errors. The SINS positioning system with the motion constraint-aided ZUPT model can follow the reference trajectory effectively. Moreover, the maximum position error with this method is 1.6 m in 180 s and increases by 92.0% and 88.1% compared with the single motion constraint model and single ZUPT model, respectively. Finally, the shearer model moves in four circles along with the track, and the experiment lasts 718 s. the experimental results show that the SINS positioning system with the motion constraint-aided ZUPT model can track the reference trajectory effectively. Meanwhile, the maximum position error with this algorithm is 2.4 m. The positioning accuracy proposed in this paper surpasses that of the single motion constraint model and the single ZUPT model, respectively. In addition, the SINS positioning system with this algorithm has better stability.

## Figures and Tables

**Table 1:** The main parameters of the MEMS accelerometer and gyroscope.

Accelerometer		Gyroscope	
Parameters (unit)	Value	Parameter	Value
Measured scale of *x*, *y*, *z* axis (*g*)	<±4	Measured scale of Rolling, pitching, heading (°/s)	<±200
Measured bias (mg)	<1	Measured bias (°/h)	<3
Nonlinearity (% FS)	<1	Nonlinearity (% FS)	<0.15
resolution ratio (mg)	<0.5	resolution ratio (°/s)	<0.025
Measured bandwidth (Hz)	25	Measured bandwidth (Hz)	25

The unit *g* is expressed as gravitational acceleration in Table 1.

**Table 2:** Performance comparison for different integration models.

Item	Axis	SINS	SINS + MC	SINS + ZUPT	SINS + MC + ZUPT
**Position error range (m)**	*x*	−4.3382 to 10.6647	−1.4900 to 2.8976	−2.3743 to 0.2435	−1.2728 to 0.4189
	*y*	−44.0027 to 23.8298	−19.3788 to 8.2045	−1.5554 to 7.5550	−1.7483 to 2.9030
**Variance**	*x*	7.9740	0.9498	0.4527	0.0341
	*y*	239.9170	40.0222	2.9617	0.7557
**Maximum (m)**		44.9933	19.5625	7.7194	1.4552
**Average (m)**		18.4780	7.6836	2.9190	0.6207
